# Cut-Offs and Response Criteria for the Hospital Universitario La Princesa Index (HUPI) and Their Comparison to Widely-Used Indices of Disease Activity in Rheumatoid Arthritis

**DOI:** 10.1371/journal.pone.0161727

**Published:** 2016-09-07

**Authors:** Isidoro González-Álvaro, Isabel Castrejón, Ana M. Ortiz, Esther Toledano, Santos Castañeda, Alberto García-Vadillo, Loreto Carmona, EMECAR Study Group

**Affiliations:** 1 Rheumatology Department, Hospital Universitario la Princesa, IIS-IP, Madrid, Spain; 2 Division of Rheumatology, Rush University Medical Center, Chicago, Illinois, United States of America; 3 Rheumatology Department. Hospital Clínico San Carlos, IdISSC, Madrid, Spain; 4 Instituto de Salud Musculoesquelética, Madrid, Spain; Peking University First Hospital, CHINA

## Abstract

**Objective:**

To estimate cut-off points and to establish response criteria for the Hospital Universitario La Princesa Index (HUPI) in patients with chronic polyarthritis.

**Methods:**

Two cohorts, one of early arthritis (Princesa Early Arthritis Register Longitudinal [PEARL] study) and other of long-term rheumatoid arthritis (Estudio de la Morbilidad y Expresión Clínica de la Artritis Reumatoide [EMECAR]) including altogether 1200 patients were used to determine cut-off values for remission, and for low, moderate and high activity through receiver operating curve (ROC) analysis. The areas under ROC (AUC) were compared to those of validated indexes (SDAI, CDAI, DAS28). ROC analysis was also applied to establish minimal and relevant clinical improvement for HUPI.

**Results:**

The best cut-off points for HUPI are 2, 5 and 9, classifying RA activity as remission if ≤2, low disease activity if >2 and ≤5), moderate if >5 and <9 and high if ≥9. HUPI’s AUC to discriminate between low-moderate activity was 0.909 and between moderate-high activity 0.887. DAS28’s AUCs were 0.887 and 0.846, respectively; both indices had higher accuracy than SDAI (AUCs: 0.832 and 0.756) and CDAI (AUCs: 0.789 and 0.728). HUPI discriminates remission better than DAS28-ESR in early arthritis, but similarly to SDAI. The HUPI cut-off for minimal clinical improvement was established at 2 and for relevant clinical improvement at 4. Response criteria were established based on these cut-off values.

**Conclusions:**

The cut-offs proposed for HUPI perform adequately in patients with either early or long term arthritis.

## Introduction

**Fig 1:**
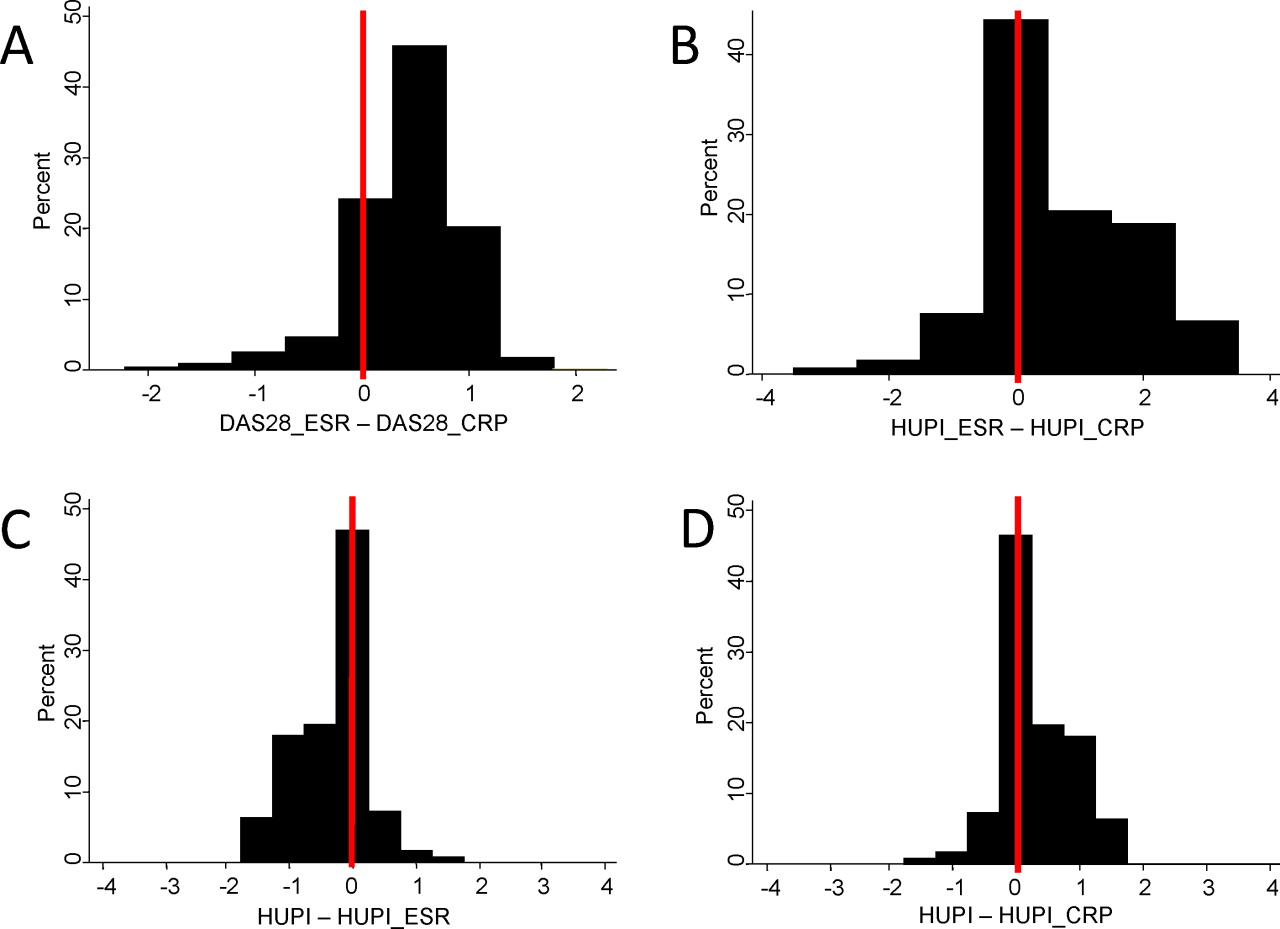
Interchangeability of disease activity score values calculated with erytrosedimentation rate (ESR) or C-reactive protein (CRP) is more accurate with HUPI than with DAS28. Data are shown as percent of visits with a given difference between calculated scores: A) DAS28 calculated with ESR (DAS28_ESR) minus DAS28 calculated with CRP (DAS28_CRP), B) HUPI calculated with ESR (HUPI_ESR) minus HUPI calculated with CRP (HUPI_CRP), C) HUPI calculated with both acute phase reactants (HUPI) minus HUPI_ESR, and D) HUPI minus HUPI_CRP. Vertical red lines show the percent of visits where the scores from both pairs was equal (Δ = 0). In the case of DAS28 a difference of ±0.25 was considered equal to null.

The treat to target (T2T) strategy has improved the outcome of rheumatoid arthritis (RA) and therefore has been included in established RA guidelines [1,2]. In relation to this strategy, there is substantial agreement on considering remission, or low disease activity, as the therapeutic objective for most patients with RA [1,2]. However, DAS28 and SDAI, the most widely-used indices for defining these targets, have several biases that may interfere with their accuracy [3–6]. DAS28 has the additional inconvenient that its values differ when it is calculated with either erytrosedimentation rate (ESR) or C-reactive protein (CRP) [7,8]. Furthermore, both DAS28 and SDAI have a gender bias derived from the larger number of tender joints and of ESR values in women compared to men [5,9,10]. To overcome these issues, we developed the Hospital Universitario La Princesa Index (HUPI), an index for the assessment of disease activity in chronic polyarthritis that includes the same domains as in DAS28 and SDAI but corrected by gender and easier to calculate than the DAS28 (Table 1) [6]. Moreover, it can be calculated with either CRP, or ESR or both. Fig 1 shows how the interchangeability of values is better with HUPI than with DAS28. This may be of special interest in observational studies to avoid missing data. HUPI has demonstrated high accuracy, feasibility and sensitivity to change, even superior to those of DAS28 and SDAI [6]. However, to be useful both in daily clinical practice and clinical trials it is necessary to define cut-off points that allow establishing different levels of disease activity, as well as response criteria, such as those based on DAS28 [11].

**Table 1:** Scoring of the variables used to calculate HUPI.

		0	1	2	3
**Tender joints/28**	♀	0	1–2	3–6	>6
	♂	0	1	2–3	>3
**Swollen joints/28**		0	1–2	3–4	>4
**GDA-Patient (0–100)**		0–15	16–30	31–50	>50
**C-reactive protein (mg/dl)**		≤0.1	0.11–0.8	0.81–1.5	>1.5
**Erythrosedimentation rate**	♀	0–15	16–20	21–30	>30
	♂	0–10	11–15	16–20	>20

GDA: global disease assessment. HUPI is calculated as the sum of four variables (graded 0–3): 28 tender and swollen joint counts, global disease assessment by physician and acute phase reactants (the average score value of ESR and CRP must be used if both acute phase reactants are considered). The score of these variables was based in their quartile distribution in the population used to describe the index [6].

The objectives of this study were 1) to establish cut-off points for HUPI able to discriminate between activity levels both in early arthritis and in long-term RA populations; 2) to establish response criteria based on HUPI; and 3) to compare the accuracy and discriminant ability to that of widely-used indices.

## Methods

### Patients

This is a validation study with repeated measures for which we used data obtained from two cohorts. HUPI was developed and validated in a mixed population of early RA and undifferentiated arthritis (UA) patients, the Princesa Early Arthritis Register Longitudinal study (PEARL) cohort [6], which we now use also to define cut-offs. In order to determine whether HUPI and its cut-offs are valid in long-term RA we added the data from the Estudio de la Morbilidad y Expresión Clínica de la Artritis Reumatoide (EMECAR) cohort [12].

The research carried out in PEARL and EMECAR is in compliance with Helsinki Declaration. The Ethics Committee for Clinical Research at Hospital Universitario de La Princesa reviewed and approved both protocols, and all patients included in both studies signed an informed consent form.

### The Princesa Early Arthritis Register Longitudinal (PEARL) cohort

PEARL includes patients recruited from the early arthritis clinic of Hospital Universitario La Princesa, Madrid (Spain). Patients with a suspicion of arthritis of less than a year since symptom onset are referred to this clinic. Patients diagnosed with gout, septic or viral arthritis, osteoarthritis, spondyloarthropathies or connective tissue diseases during the first 2 years of follow-up are excluded from the cohort. Therefore, for this specific analysis, only patients fulfilling the 1987 ACR RA criteria [13] or with chronic undifferentiated arthritis (UA) were included. The clinical protocol includes five visits during a follow-up period of 5 years (baseline, 6, 12, 24 and 60 months) in which demographic, clinical, therapeutic, and laboratory data as well as biologic samples are collected. With the information collected it is possible to estimate the value of the following disease activity indexes: DAS28-ESR [14], DAS28-CRP (calculated as described in *http://www.das-score.nl*), SDAI [15], CDAI [15] and HUPI [6]. More detailed information about the protocol has been described previously [16].

### The EMECAR cohort

EMECAR was a prospective longitudinal cohort of prevalent RA patients selected by random sampling in 34 Rheumatology Units from Spain. The follow-up took place from November 1999 to December 2004. EMECAR database includes the required information to calculate DAS28-ESR, DAS28-CRP and HUPI, but not SDAI or CDAI since global disease assessment by physician was not collected. Remission was defined in EMECAR by the Pinal’s criteria [17]. A more detailed description of the EMECAR cohort has been published previously [12].

### Definition of low, moderate and high disease activity

We used two definitions, depending on the cohort.

Low, moderate and high disease activity levels were established in a subset of PEARL as previously reported [18]. Six experienced rheumatologists (AMO, ET, IC, SC, AG-V and IG-A) categorized the level of disease activity into low-moderate-high at each visit based on the following information: 28 tender and swollen joint counts, global disease assessment by patient in a 0 to 100 mm scale, Health Assessment Questionnaire, ESR, and CRP. The assessment was retrospective and therefore, the physicians could not physically examine the patients. In addition, they were blinded to the global disease assessment by physician obtained on a 0 to 10 visual analog scale at each visit.

In EMECAR, low, moderate and high disease activity level were defined by the DAS28-ESR classical cut-offs (2.6, 3.2 and 5.1) [14].

### Criteria to define remission

Considering that at present there is no definitive consensus on a best definition for remission and the differences in data collection at the two cohorts, we used 4 operational definitions to estimate this cut-off point for HUPI: 1) by the consensus of the 6 experienced rheumatologists in the subpopulation of PEARL; 2) by the ACR/EULAR remission criteria according to SDAI (<3.3); 3) by the ACR/EULAR remission criteria according to Boolean criteria [19]; and by the criteria by Pinals et al [17].

### Estimation of cut-off points

Receiver operating characteristic (ROC) analysis was performed using the “roctab” command of Stata 12.1^®^ for Windows (StataCorp LP, College Station, TX, USA). Each cut-off point was selected on the basis of the best trade-off values between sensitivity and specificity. ROC curves were also obtained with the “roctab” command of Stata, using the “graph” option. To estimate whether the differences in the area under the curve (AUC) between the indices were statistically significant, we used the Stata “roccomp” command that provides a test for the equality of the AUCs using an algorithm described by DeLong et al.[20]. Statistical significance was accepted if the p value was lower than 0.05.

### Estimation of minimal and relevant clinical improvement for HUPI

PEARL patients whose ΔDAS28-ESR between baseline and 6 months follow-up visits was ≥0.6 were considered to experience, at least, a minimal clinical improvement. Those patients whose ΔDAS28-ESR was ≥1.2 were considered to experience a relevant clinical improvement.

The best value for minimal or relevant clinical improvement for HUPI was selected on the basis of the best trade-off values between sensitivity and specificity from the respective ROC curves as described above for cut-off points to separate disease activity levels.

## Results

### Patients’ characteristics

We studied data from 1547 visits of 403 patients included in PEARL study and 2880 visits belonging to 789 patients from EMECAR. Since the former is an early arthritis register and the later a prevalent RA cohort, patients from PEARL study were on average 6 years younger than EMECAR patients (Table 2). Accordingly, at baseline, disease duration was longer in EMECAR patients but slightly higher disease activity was observed in patients from PEARL study (Table 2), since these patients were untreated at baseline visit whereas EMECAR patients were under treatment when enrolled at the cohort. Table 2 also shows the number of visits in which the patients were at remission, low, moderate or high disease activity (defined by DAS28-ESR cut-off points: 2.6, 3.2, 5.1).

**Table 2:** Baseline characteristics of patients and distribution of disease activity level in all visits from studies PEARL and EMECAR.

	EMECAR	PEARL
Number of patients	789	403
Age, m ± SD	61 ± 13	55 ±16
Female gender, n (%)	568 (72)	322 (80)
Disease duration (years; IQR)	8.7 [4.1–12.6]	0.5 [0.3–0.7]
RA / UA, n (%)	789 (100) / -	280 (69.5) / 123 (30.5)
RF positive, n (%)	592 (75)	208 (51.6)
DAS28-ESR, mean ± SD	4.2 ± 1.4	4.5 ± 1.5
DAS28-CRP, mean ± SD	-	4.0 ± 1.4
SDAI, median [IQR]	-	17.0 [8.9–32.1]
CDAI, median [IQR]	-	14.8 [8.1–30.1]
HUPI, mean ± SD	6.5 ± 2.9	7.0 ± 3.2
Visits	2,880	1,547
Disease activity level defined by DAS28-ESR. N (%)		
Remission	560 (19.4%)	438 (28.3%)
Low	426 (14.8%)	241 (15.6%)
Moderate	1,303 (45.2%)	568 (36.7%)
High	526 (18.3%)	261 (16.9%)
No DAS28-ESR available	65 (2.3%)	39 (2.5%)

EMECAR: *Estudio de la Morbilidad y Expresión Clínica de la Artritis Reumatoide*; PEARL: Princesa Early Arthritis Register Longitudinal; IQR: interquartile range; RA: rheumatoid arthritis; UA: undifferentiated arthritis; RF: rheumatoid factor; DAS28-ESR: disease activity score calculated with erythrosedimentation rate; DAS28-CRP: disease activity score calculated with C-reactive protein; SD: standard deviation; SDAI: simplified disease activity index; CDAI: clinical disease activity index; HUPI: Hospital Universitario Princesa Index.

### Cut-off points for low/moderate and moderate/high disease activity

**Fig 2:**
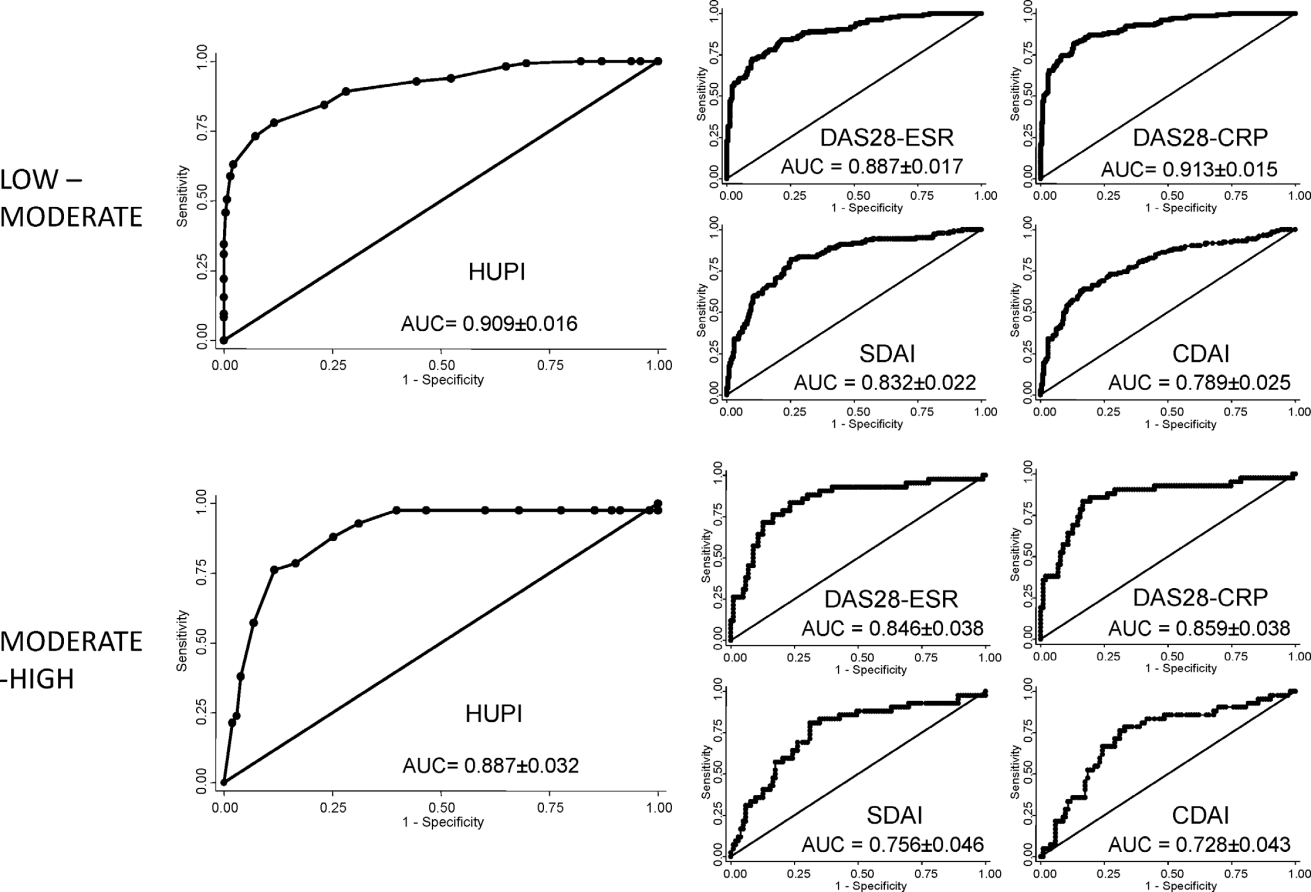
HUPI and DAS28 discriminate low-moderate and moderate-high disease activity better than SDAI and CDAI. Receiver operating curves were plot using the “roctab” command of STATA, with the option graph. Data obtained from the PEARL cohort. The best cut-off point to discriminate between moderate and high disease activity was 9 (Table 3). The AUC of HUPI was systematically larger than those from the other indices (Fig 1 lower panels), although differences were only significant with SDAI (p = 0.0004) and CDAI (p = 0.0001).

The best threshold value of the HUPI to discriminate between low and moderate disease activity was 5 (Table 3). As it is shown in Fig 2 (upper panels), there were no significant differences between area under ROC curves of HUPI and the two versions of DAS28. However, the AUC of HUPI was significantly greater than those of SDAI (p = 0.0002) and CDAI (p<0.0001). Likewise, both DAS28 versions (ESR and CRP) showed higher ROC curves areas than SDAI and CDAI (p<0.0001).

**Table 3:** Cut-offs for low-moderate and moderate-high disease activity in HUPI.

Population	Disease activity	AUC	Cut-off point	Se	Sp	LR +	LR -
PEARL	Low—Moderate	0.909	>5	72%	89%	3.18	0.15
EMECAR	Low—Moderate	0.945	>5	81%	92%	10.03	0.21
PEARL	Moderate–High	0.887	≥9	72%	90%	3.24	0.14
EMECAR	Moderate–High	0.937	≥9	70%	93%	10.45	0.32

HUPI: Hospital Universitario Princesa Index; AUC: area under curve; Se, sensitivity; Sp, specificity; LR: likelihood ratio EMECAR: Estudio de la Morbilidad y Expresión Clínica de la Artritis Reumatoide; PEARL: Princesa Early Arthritis Register Longitudinal.

### Remission cut-off point for HUPI

Table 4 summarizes the best cut-off points for remission considering the different populations and definitions described under Methods. In view of all these results, we decided to choose 2 as cut-off for remission in HUPI.

**Table 4:** Cut-off points for remission in HUPI.

Population	Remission definition	AUC	Cut-off point	Se	Sp	LR +	LR -
PEARL	Rheumatologists consensus	0.962	≤2	84%	92%	5.74	0.09
PEARL	ACR/EULAR SDAI<3.3	0.976	≤2	90%	93%	9.66	0.07
PEARL	ACR/EULAR Boolean	0.949	<2	88%	92%	7.57	0.09
EMECAR	Pinals criteria	0.890	≤2	49%	95%	1.86	0.11

HUPI: Hospital Universitario Princesa Index; AUC: area under curve; Se: sensitivity; Sp: specificity; LR: likelihood ratio; PEARL: Princesa Early Arthritis Register Longitudinal; EMECAR: Estudio de la Morbilidad y Expresión Clínica de la Artritis Reumatoide; ACR: American College of Rheumatology; EULAR: European League Against Rheumatism; SDAI: simplified disease activity index.

**Fig 3:**
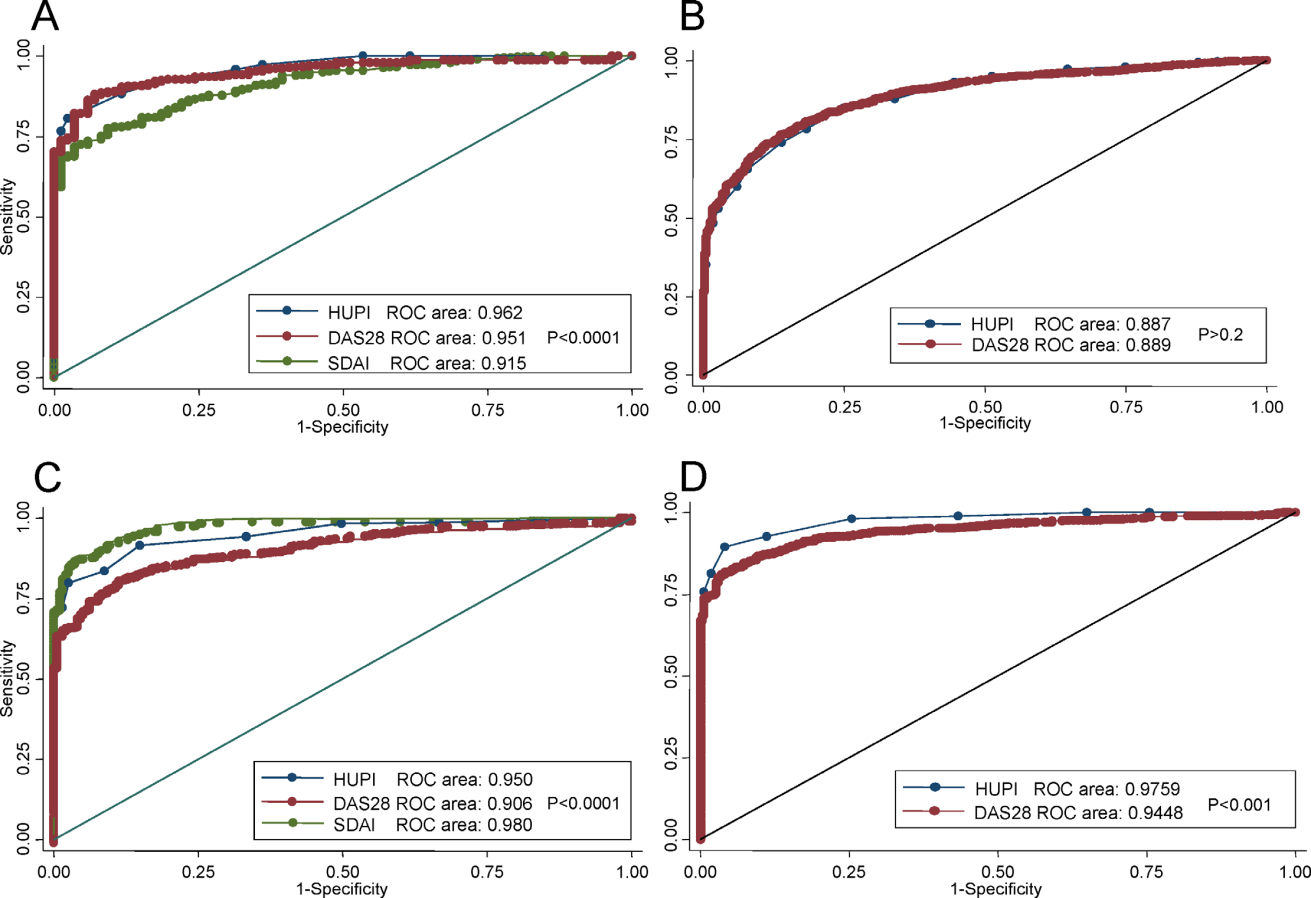
Receiver operating curves (ROC) for HUPI, SDAI and DAS28 to discriminate remission. A) Remission defined by rheumatologist consensus in PEARL population. B) Remission defined by Pinals criteria in EMECAR population. C) Remission defined by ACR/EULAR boolean criteria in PEARL population. D) Remission defined by ACR/EULAR SDAI<3.3 criteria in PEARL population. Receiver operating curves were plot using the “roccomp” command of STATA, with the option graph. This command also tests the equality of ROC areas.

In order to determine whether HUPI is more accurate than DAS28 or SDAI for estimating remission, we compared DAS28 and HUPI in EMECAR and PEARL with all previous definitions and SDAI and HUPI in PEARL with rheumatologist assessment and Boolean based ACR/EULAR remission criteria. As it is shown in Fig 3, HUPI is, depending on definition of remission, as accurate as DAS28 (A and B panels) or significantly superior (ACR/EULAR remission definitions at C and D panels). HUPI was significantly better than SDAI when using the classification of remission by experienced rheumatologists (Fig 3A) but worse than SDAI when using the 2011 Boolean definition of remission (Fig 3C).

**Fig 4:**
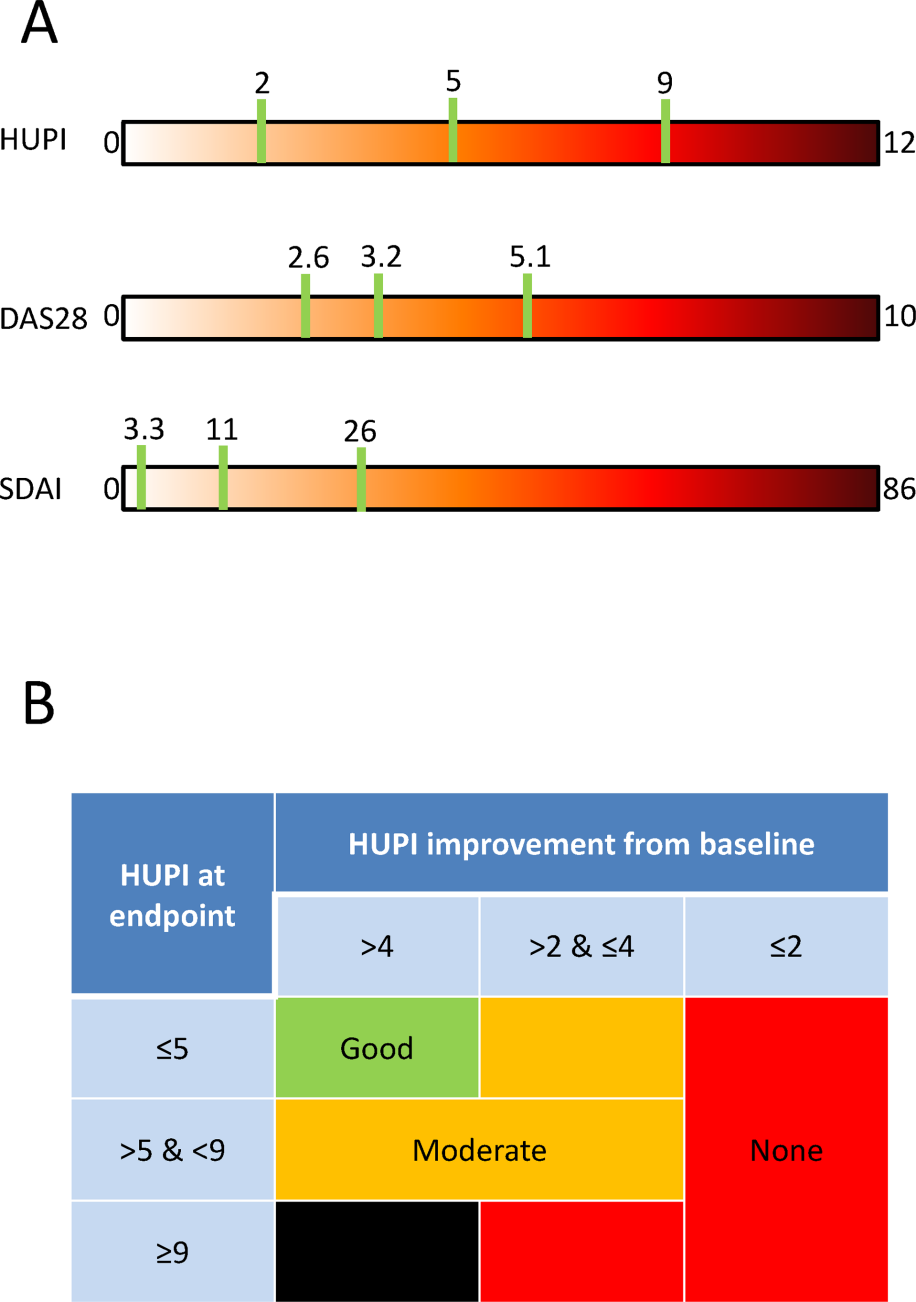
Cut-off points and response criteria for HUPI. A) Comparison of cut-off points’ distribution along HUPI, DAS28 and SDAI’s range of values. B) Response criteria using HUPI based on its cut-off values for low, moderate and high disease, as well as minimal and relevant improvements. The black box means that in no case the HUPI can be ≥9 if the improvement in HUPI score is ≥4.

Fig 4A shows how cut-off points to define remission, low, moderate and high disease activity are distributed along the range of values of DAS28, SDAI and HUPI. Interestingly, the distribution of the cut-off points on the HUPI is more homogeneous in HUPI than in the other scales.

### Response criteria using HUPI

A minimal clinical improvement in HUPI, corresponding to a 0.6 point improvement in DAS28, was 2 (Sensitivity [Se] 73%, Specificity [Sp] 84%; Positive likelihood ratio [LR+] 3.15, LR- 0.21). A relevant clinical improvement in HUPI, corresponding to a 1.2 points improvement in DAS28, was 4 (Se 65%, Sp 90%; LR+ 6.22, LR– 0.39). Considering the cut-off points and the estimation for relevant improvement for HUPI, Fig 4B shows how to classify the response into none, moderate or good by using the HUPI.

## Discussion

The assessment of disease activity in RA through composite indexes, such as DAS28 or SDAI, has been a cornerstone in clinical research, translational research and daily clinical practice. However, several concerns affect both indexes: a difficult calculation of DAS28, gender bias, left skewed distribution of their values leading to low sensitivity to change, especially with SDAI [4–6,21]. All these problems led us to develop HUPI, with the hope that it could be useful in clinical and translational research and probably in daily clinical practice since it is easy to calculate without calculator. However, to be useful in the current T2T strategies we needed specific cut-off values to determine the common targets (clinical remission or low disease activity) accepted at present, and this study has established them.

In addition, the present study suggests that HUPI is more accurate than SDAI to discriminate low-moderate disease activity. This is an important landmark of HUPI since this level of activity is commonly used to prescribe biological therapy [1,2] as well as to initiate tapering of kind of treatment [22].

Furthermore, depending on the definition, HUPI discriminates clinical remission better than DAS28. This may be due to the fact that HUPI corrects for gender and for extreme values, both accepted as interfering with the definition of remission [23].

One of the main limitations of this study is the absence of real gold standards for evaluating disease activity. This was the reason leading to the use of several definitions of remission and different ways of defining low, moderate and high disease activity either in PEARL and EMECAR. Nevertheless, the results in both populations were quite concordant, which is reassuring on the validity of the HUPI. Another limitation of HUPI is that it may suffer from a ceiling effect in patients with very high disease activity. It is possible that in clinical trials in which such kind of patients is preferentially included sensitivity to change might be more difficult to demonstrate.

We are aware that implementation of HUPI will be difficult since DAS28 and SDAI are deeply accepted by rheumatologists. However, we provide this information convinced that HUPI will be useful to scientists involved in RA clinical and translational research. It would be especially interesting when needing complex multivariable analysis since HUPI suffers from less biases than DAS28 and SDAI and its values show a normal distribution.

Finally, further validation of the index, its cut-offs and response criteria will be needed in different cohorts, either in early and long term RA populations, as well as in patients with very high disease activity from clinical trials. Of special interest will be to study whether HUPI or current validated indexes explain better radiological progression or biological processes.

In summary, we have estimated the most adequate cut-offs for the HUPI to define levels of activity in RA (low-moderate-high and remission), which are stable in early and long-term populations, and found that HUPI is at least as good as widely established indices.

## Supporting Information

S1 File Data from PEARL study used in this article are provided. (XLS)
